# The Posterior Atlantooccipital Membrane: The Anchor for the Myodural Bridge and Meningovertebral Structures

**DOI:** 10.7759/cureus.25484

**Published:** 2022-05-30

**Authors:** Frank Scali, Ai Ohno, Dennis Enix, Sherif Hassan

**Affiliations:** 1 Medical Education and Anatomy, California University of Science and Medicine, Colton, USA; 2 Medicine, California University of Science and Medicine, Colton, USA; 3 Research, Independent Investigator, Ballwin, USA

**Keywords:** rectus capitis posterior, posterior atlantooccipital membrane, obliquus capitis, myodural bridge, atlantoaxial interspace

## Abstract

Introduction: Sheet plastination has provided evidence that the posterior atlantooccipital membrane attaches to the dura’s posterior sleeve at the cerebrospinal junction. These findings contradict the traditional anatomical description of this membrane extending from the atlas’ posterior arch to the foramen magnum.

Methods: A total of 16 plastinated cadavers were studied to evaluate the *in situ *and gross configuration of the posterior atlantooccipital membrane. Fifteen cadavers underwent sheet plastination, and one head was hemisected and plastinated. In all specimens, stereomicroscopy was used to evaluate the posterior atlantooccipital membrane and related structures within the intervertebral and epidural spaces.

Results: In all 16 specimens, the posterior atlantooccipital membrane extending from the occiput, merged with the craniocervical dura mater, and formed a membrane-dura complex that ended at the level of the third cervical vertebra. The superior and inferior myodural bridge coalesced with their respective vertebrodural ligaments and fused with the posterior atlantooccipital membrane at their respective interspaces.

Conclusion: The median aspect of the posterior atlantooccipital membrane does not directly communicate with the posterior arch of the atlas. Instead, the posterior atlantooccipital membrane converges with the craniocervical dura mater and terminates at the level of the third cervical vertebra. This membrane-dura complex serves as a common attachment site for the myodural and vertebrodural structures.

## Introduction

The cervical epidural space contains an intricate network of fascial and ligamentous structures that communicate with the dura mater [1-9]. These so-called meningovertebrodural connections have been verified by conventional dissection techniques, histology, immunohistochemical staining, magnetic resonance imaging, sheet plastination, and confocal microscopy [3-7,9-11]. Over the last decade, these bridging connections have been identified across a wide variety of species in the animal kingdom and today are considered non-incidental universal structures [12,13].

The relationship between the myodural structures and the posterior atlantooccipital membrane (PAOM) is rarely discussed. Traditionally, the PAOM has been defined as a membrane extending from the superior border of the posterior atlantal arch to the foramen magnum’s posterior margin [3,14-16]. Nash et al. (2005) demonstrated that the superior myodural bridge (MDB) communicates with the cervical dura mater indirectly via the PAOM [10]. Scali et al. in 2015 described an analogous communication between the inferior MDB and the PAOM. This latter group of authors noted that the PAOM extended along the posterior surface of the dura mater, ending at the level of C3 [9]. Despite the evidence demonstrated by these two teams of researchers, the PAOM continues to be erroneously defined as a dense band extending directly from the posterior arch of the atlas to the foramen magnum [8,17].

Understanding the PAOM’s relationship with upper cervical myovertebral structures is essential when discussing the etiology of cervicogenic headaches and neck pain. Excessive tension across the myodural structures influences pain-sensitive dura via the PAOM [9,18]. The PAOM’s anatomy is also encountered during routine suboccipital craniectomy procedures (Chiari I decompression) [15]. To alleviate symptoms of syringomyelia, the PAOM is incised. Adhesions may form along the PAOM, resulting in constriction at the craniocervical junction leading to complications and recurrence of symptoms [16].

The configuration of the PAOM and its related connections requires clarification. This study uses E12 plastination to investigate the PAOM’s communication with the craniocervical dura mater, myodural bridging structures, and related vertebrodural ligaments.

## Materials and methods

A total of 16 donor specimens were used in this study. Fifteen specimens (12 males and three females, ages 67-89 years) were prepared for sheet plastination. One hemisected head underwent prosection, plastination, and post-plastination dissection. Initial prosection and plastination were prepared and obtained from Prime Prosections. Post-plastinated dissection was conducted at the California University of Science and Medicine. Post-plastination dissection was performed by an anatomist with 15 years of prosection experience. A second anatomist with 26 years of experience performed a quality assessment and confirmed the findings. Specimens with signs of suboccipital variations, prior surgery, or trauma in the region of interest were excluded from this study. Measurements were recorded using a Neiko 01407A Electronic Digital Caliper (range: 0-150 mm; resolution 0.01 mm; accuracy ± 0.02 mm; China: Neiko Tools). Photographs were captured with a Nikon D40 Digital Camera (Tokyo, Japan: Nikon Corporation). All guidelines for the use of cadaveric material were followed.

Plastination

All 16 specimens underwent E12 plastination technique [19]. Fifteen out of the 16 specimens underwent serial sagittal sectioning before plastination. Preparation of sheet sections required embalming of cadaveric specimens followed by freezing at −85°C for 48 hours. Sheet plastination sections varied from 2 mm to 4 mm in thickness. One mid-sagittally hemisected head underwent standard prosection and followed methods for additional preservation using the identical E12 plastination procedure.

Following initial slicing or prosection, all specimens were dehydrated and degreased by immersing tissue into acetone chilled at −25°C. Acetone concentration was gradually increased for 20-22 weeks until the dehydration and degreasing process was approved based on tissue clarity, shrinkage, and preservation. Next, vacuum impregnation of resin mixture was conducted under cryogenic conditions (−8 to 0°C) over two days. Impregnation was complete when bubbles failed to appear from tissue specimens. Sections were then laminated by being placed in a warm water bath and then in an oven set at 35°C for 24 hours to achieve solidification.

Translucent sheet plastinates were examined on a radiographic light-emitting diode lightbox. Each slice was subsequently reviewed macroscopically under low magnification (range: 0.63× to 1.25×) with a Leica MZ8 Stereoscopic Dissecting Microscope (Buffalo Grove, IL: Leica Microsystems Inc.). Structures within the intervertebral and epidural spaces were measured and photographed.

Post-plastination dissection

The PAOM was identified and prosected under a Leica A60F stereomicroscope (range: 5× to 30×; Buffalo Grove, IL: Leica Microsystems Inc.). Under 20× to 30× magnification, E12 polymer surrounding the PAOM was identified by its characteristic grey-white translucent appearance and was separated from biological tissues using microdissection instruments. Polymer from between fascial layers was excised down to the level of C3-C4. Excised polymer was further examined under 30× magnification to ensure biological tissue was removed from the specimen. All bridging and ligamentous structures forming attachments with the PAOM were traced to its origin, identified, and then excised to clarify the PAOM’s continuity. The thickness of the PAOM was measured and photographed.

## Results

Sheet plastination

**Figure 1:**
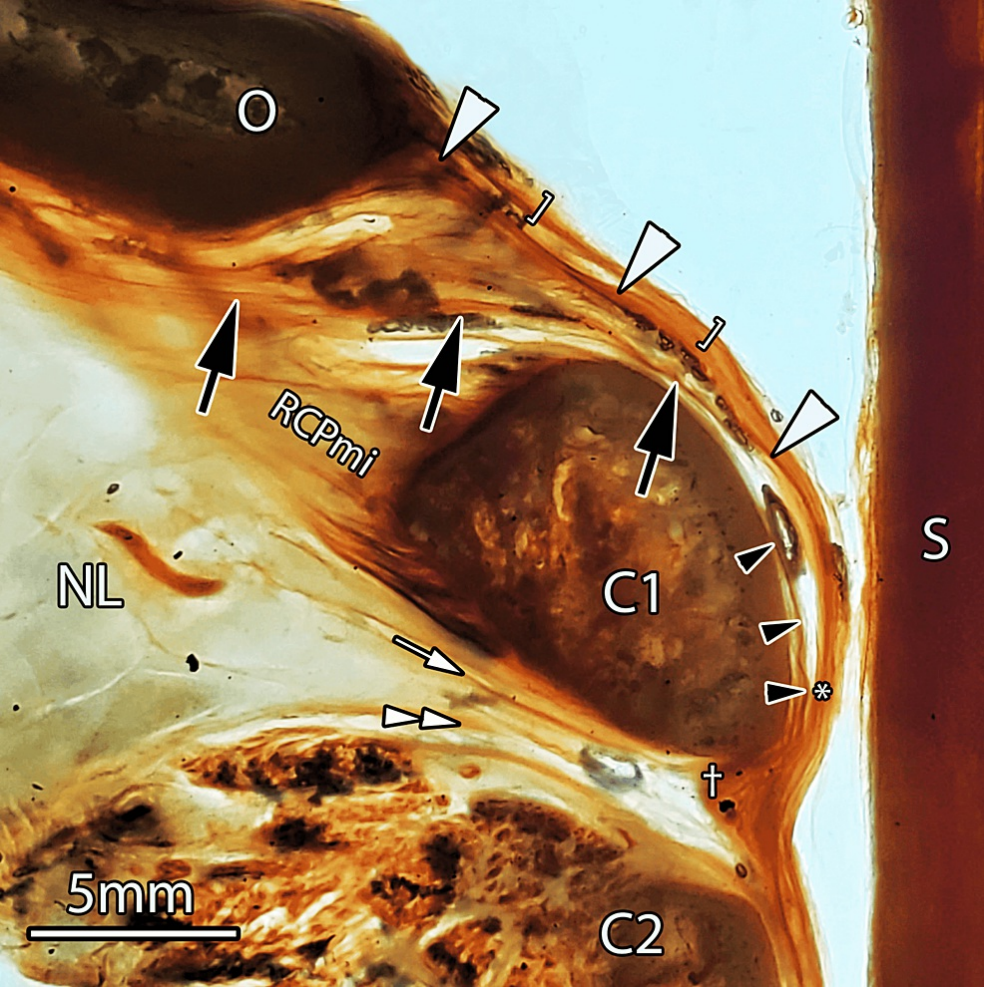
The atlantooccipital and atlantoaxial interspaces in a midsagittal sheet plastinated slice measuring 4 mm in thickness. The PAOM (white arrowheads) extends from the occiput (O) and merges with the dura mater (brackets). The PAOM does not attach to the atlas (C1). Instead, the superior myodural bridge (black arrows) coalesces with the PAOM (*). Scale bar = 5mm, inferior myodural bridge (white arrow), nuchal bridge (double white arrowheads), superior vertebrodural ligament (black arrowheads), and atlantoaxial ligamentum flavum (†). O: occiput; C1: atlas; C2: axis; S: spinal cord; RCPmi: rectus capitis posterior minor; NL: nuchal ligament; PAOM: posterior atlantooccipital membrane; LF: ligamentum flavum

The same fibrous connective tissue configurations were found in all fifteen samples, with only minor individual variations. Periosteum extended from the superior and inferior ridge of the foramen magnum’s posterior border. These two layers converged and passed anteroinferiorly into the spinal canal. As a single entity, this band of tissue was identified as the PAOM. The craniocervical dura mater lined the PAOM’s superior border. Within the craniocervical junction, the PAOM and dura mater appeared as a single membrane-dura (membranodural) complex. This membranodural complex continued inferiorly alongside the posterior sleeve of the dura mater. Within the atlantooccipital interspace, two distinct layers of tissue circumvented the superior border of the atlas’ posterior arch to enter the epidural space. The inferior layer was traced to the rectus capitis posterior minor (RCPmi) and identified as the superior MDB. The origin of the second layer could not be identified. Both layers merged with the vertebrodural ligament of C1 (superior vertebrodural ligament) and fused with the membranodural complex within the epidural space but did not contribute to its thickness (<0.10 mm). Two additional layers of tissue were noted, passing anteroinferiorly towards the membranodural complex at the level of the atlantoaxial interspace. The superior layer was identified as the atlantoaxial (inferior) MDB, and the inferior layer was traced to the nuchal ligament and identified as the nuchal bridge. The ligamentum flavum was noted between the cervical vertebrae (Figure 1). 

Post-plastination dissection

**Figure 2:**
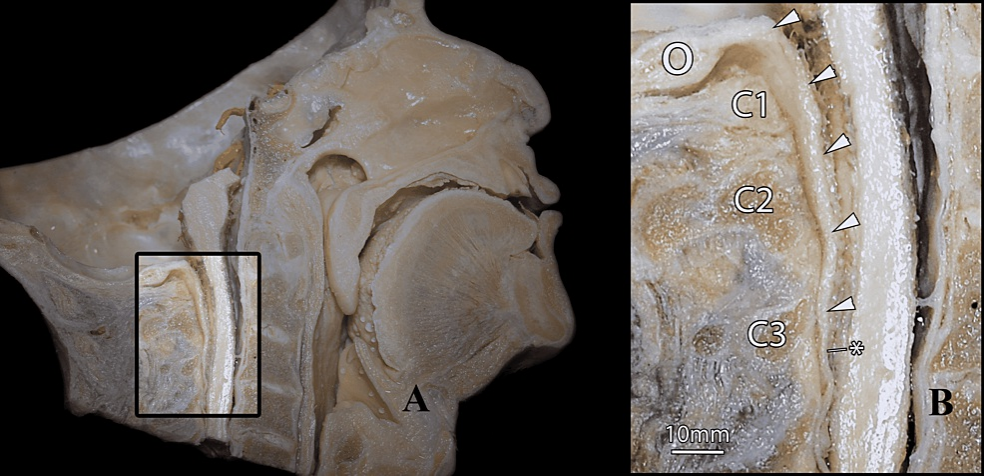
A mid-sagittal hemisected adult head that has undergone plastination and post-plastinated dissection. (A) Anatomy of the epidural contents is noted down to the level of the fifth cervical vertebra. Excess resin, superior and inferior myodural bridge, nuchal bridge, and vertebrodural ligaments were excised from this specimen under microscopy to clarify the PAOM's morphology and its relation to the dura mater. An area of interest is highlighted as a call-out. (B) Magnified view of the call-out. The PAOM extends from the occiput's anterior border, passes anteroinferiorly, and merges with the cervical dura mater in what appears to be a single band of tissue (arrowheads). This posterior membranodural complex measures 5mm in thickness at the atlantoaxial interspace and was easily separable using forceps. At the level of the third cervical vertebra, the PAOM ends (*), and the dura mater continues along its course within the vertebral canal. At the level of the third cervical vertebra, the thickness of the dura mater measures 2.5 mm. Scale bar = 10mm. O: occiput; C1: atlas; C2: axis; C3: third cervical vertebra; PAOM: posterior atlantooccipital membrane

The PAOM began as two layers - a superior and inferior extension of the periosteum covering the foramen magnum’s posterior border. These two bands of connective tissue converged, fused, and extended anteriorly into the foramen magnum. The thickness of this band measured 3.41 mm (± 0.02 mm) at the base of the occiput. Considering its origin, morphology, and gross appearance, this structure was identified as the PAOM. The PAOM immediately passed anteriorly to converge with and attach to the occipitocervical dura mater forming a membranodural complex with a thickness measuring 2.35 mm (± 0.02 mm) at the level of the atlantooccipital interspace. As this membranodural complex folded inferiorly into the vertebral canal, it did not directly contact the atlas. Instead, it coursed into the vertebral canal with the dura mater. The atlantooccipital (superior) and atlantoaxial (inferior) myodural and vertebrodural structures appeared to insert into the PAOM at their respective intervertebral levels as the superior and inferior meningomyovertebral ligaments. At the level of the atlantoaxial interspace, the thickness of this complex measured 2.61mm (± 0.02mm). This membranodural structure remained at this thickness along its course, then gradually tapered off (1.60mm {± 0.02 mm}) at the level of the C2-C3 junction. The PAOM appeared to end at the third cervical vertebral level, and the dura mater continued as a separate entity. The thickness of the dura mater at the level of C3 was measured at 0.90 mm (± 0.02 mm) (Figure 2). The PAOM was firmly attached to the dura mater along its length but was separable by forceps.

## Discussion

Plastination is a method of preservation that can be used to identify fascial structures in situ [19]. During the process of plastination, soft tissue structures shrink, allowing for easy visualization of fascial planes that are otherwise lost by standard dissection measures [9,10,20]. Over the past few decades, plastination has offered valuable insight into detailing the in situ arrangement of soft tissue connections extending from musculoskeletal structures to the cervical dura mater [2,8,9].

This study also used post-plastination dissection under stereomicroscopy in one specimen to evaluate and confirm findings that are otherwise difficult to dissect. During the plastination procedure, the E12 polymer infuses, expands, then congeals within the small spaces separating layers of tissue- the shrinking of soft tissue aids this process. Regions of anatomy or spaces that are difficult to visualize will separate and become easily identifiable as a translucent gray-white solid under low magnification. In their anatomical study, Pimenta et al. reported the absence of the PAO and atlantoaxial membranes in eight unembalmed adult human cadavers [21]. The results in Pimeta et al.’s study may be due to the inability to identify potential spaces when dissecting formalin-based cadavers. Post-plastination dissection under stereomicroscopy in our study allowed a prosector to identify and excise solidified polymer within the confines of potential space. This permitted the separation of tissues that are otherwise missed or damaged during conventional dissection measures.

**Figure 3:**
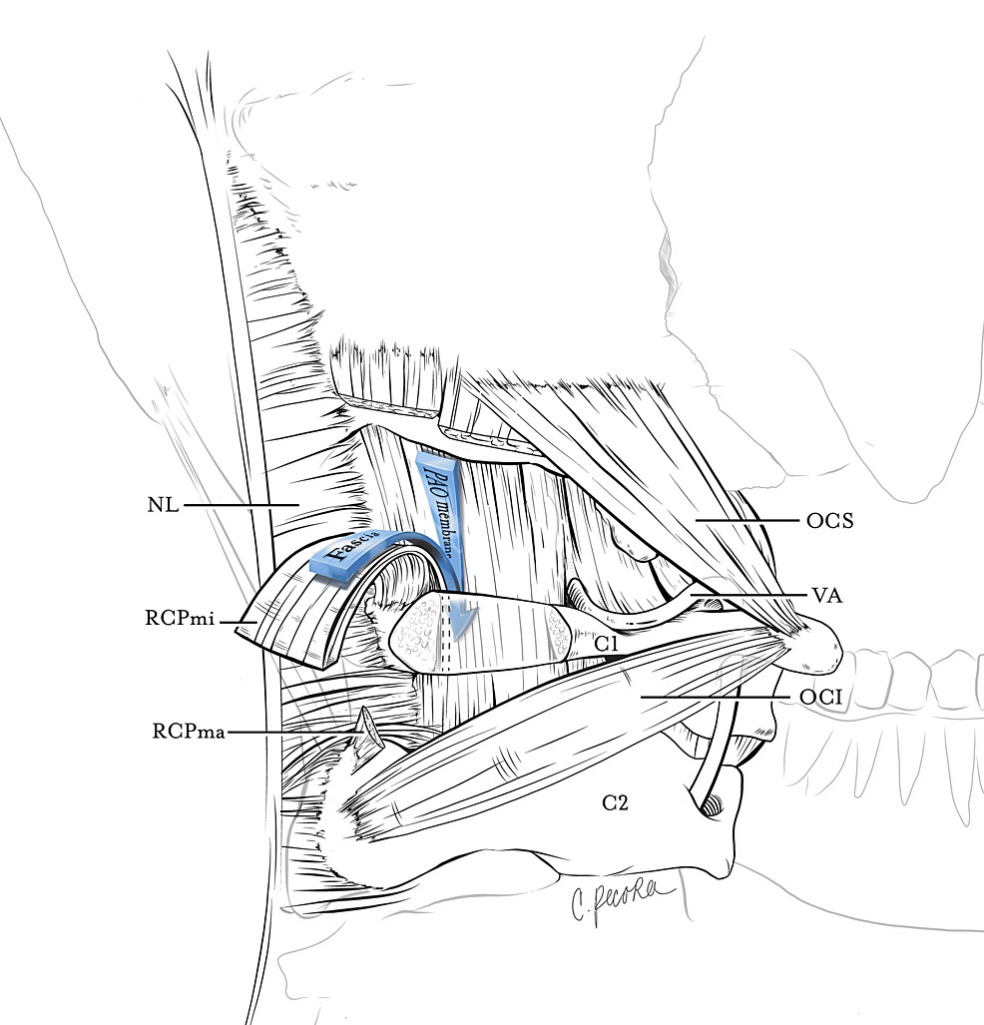
Line-art depicting the relationship between the RCPmi fascia (superior MDB) and PAOM. The RCPmi muscle is excised from its osseous insertion at the inferior nuchal line and is reflected posteriorly. The anterior fascia of the RCPmi converges with the median fibers of the PAOM at the superior boundary of the atlas (C1). This morphology creates the "illusion" of the PAOM forming a barrier at the atlantooccipital interspace. This relationship also clarifies how the superior MDB breaches the atlantoaxial interspace to communicate with the dura mater indirectly. C1: atlas; C2: axis; RCPmi: rectus capitis posterior minor; RCPma: rectus capitis posterior major; OCS: obliquus capitis superior; OCI: obliquus capitis inferior; NL: nuchal ligament; VA: vertebral artery; PAOM: posterior atlantooccipital membrane The image is an original artwork produced by Christina Pecora, MSMI, and she has given permission to use it in the study.

This study’s combined techniques confirm that the PAOM extends from the occiput, merges with the craniocervical dura mater, and ends at the third cervical vertebral level. These findings are consistent with previous studies that described the anatomical arrangement of the PAOM [9,10]. The superior MDB enters the C0-C1 interspace in an anteroinferior direction. Plastination technique reveals that no direct communication exists between the median PAOM and the posterior arch of C1. This morphology allows for access of the superior MDB into the vertebral canal (Figure 3). These findings contradict conventional descriptions that the PAOM is a continuous band extending from the atlas’ posterior arch to the foramen magnum [3,14-16].

**Figure 4:**
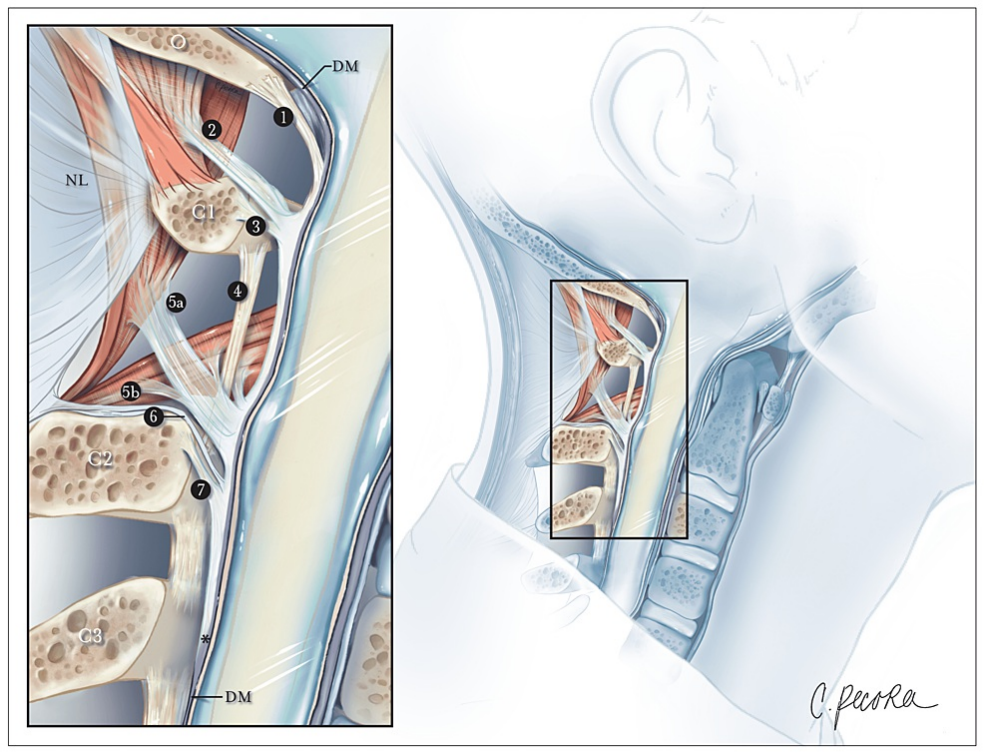
Illustration depicting the region of superior cervical attachments and ligamentous insertions in a mid-sagittal hemisected head. The posterior atlantooccipital membrane (1) extends from the occiput and coalesces with the dura mater at the cerebrospinal junction. The superior myodural bridge (2) merges with the superior vertebrodural ligament (3) of the atlas and fuses with the PAOM at the level of the atlantooccipital interspace. The inferior myodural bridge comprised of the rectus capitis posterior major fascia (5a) and obliquus capitis inferior fascia (5b) courses between the atlantoaxial ligamentum flavum (4) as bundles of dense fibers. The inferior myodural bridge fuses with the PAOM. The nuchal bridge (6) merges with the inferior vertebrodural bridge (7) and attaches to the PAOM. The PAOM terminates at the level of C3 after this transition point (*). The dura mater (DM) continues as an independent structure after that. O: occiput; C1: atlas; C2: axis; C3: third cervical vertebra; NL: nuchal ligament; PAOM: posterior atlantooccipital membrane The image is an original artwork produced by Christina Pecora, MSMI, and she has given permission to use it in the study.

The correct description of the PAOM is important as it is a universal site for upper cervical myovertebral attachments [9]. The fascia of the RCPmi forms the superior MDB, which enters the C0-C1 interspace in an anteroinferior direction [3,9,10,22,23]. As the superior MDB breaches the epidural space, it coalesces with the superior vertebrodural ligament. The superior MDB and superior vertebrodural ligament fuse with the PAOM, which attaches to the dura mater [9,11]. Fascia from the rectus capitis posterior major (RCPma) and obliquus capitis inferior (OCI) have analogous band-like communications (inferior MDB) that merge with the vertebrodural ligament of C2 (inferior vertebrodural ligament) and fuse to the PAOM at around the level of the atlantoaxial interspace (Figure 4) [9].

This layering of the myovertebral structures onto the PAOM’s posterior surface may prevent a single fascial or ligament structure from influencing the dura and its contents. From a physiological perspective, the PAOM may receive input from individual vector forces generated by dural communicating structures. The summation of these forces would then be transmitted across the PAOM and onto the dura mater influencing its subdural contents. Therefore, the PAOM may act as an “anchoring membrane” for individual structures that indirectly communicate with dura mater from the cerebrospinal cistern down to C3. This postulation aligns with a previous study that suggested transmission of force to the dura when the PAOM is stretched beyond its paraphysiological limit [24]. Evidence of this complex system having myotatic reflexive properties is supported by previous studies reporting a higher-than-expected concentration of spindle fibers in the myodural muscles, neural tissue within the myodural bridging structures, and variation of elastin tissue found at specific attachment sites along the cervical dura mater [4,6,9,25,26]. The upper cervical spine’s dura mater comprises three different tissue types: Golgi tendon organ embedded fascia, periosteal ligaments containing nociceptive fibers, and elastin-rich dura mater [25,26]. When incorporating the dense concentration of spindle fibers within suboccipital muscles and evidence of neurons embedded in the MDB, this area of anatomy constitutes a remarkably intricate network of tissue subtypes whose physiological role warrants further investigation.

The function of the MDBs and meningovertebral ligaments is still under investigation. However, researchers agree that these communications may influence the dura mater’s tension at the cerebrospinal cistern [3,5,6,9,11,27]. The PAOM’s communication with the dura mater provides further insight into these structures’ biomechanical function. During head and neck flexion, the atlantooccipital interspace widens, pulling the PAOM taut to prevent cervical dura mater enfolding [9,28]. During extension of the craniocervical joint, the RCPmi and RCPma muscles contract, creating tension along the superior and inferior myodural fascial structures, resulting in posterior traction of the PAOM [9]. The tensional force transmitted through this posterior membranodural structure may prevent dural buckling during flexion and extension at the craniocervical joint, thereby protecting against spinal cord compression [3,29]. This study also provides evidence that the PAOM contributes to the dura mater’s thickness at the cerebrospinal junction, which tapers down to the C3 vertebral level. Rotation at the atlantoaxial joint would wring or twist the dura at the upper cervical levels. A thickened layer covering the dura mater’s posterior aspect may provide added protection against dural enfolding, conserving patency at the cerebrospinal cistern during these head and neck movements. The RCPma and OCI muscles contract during atlantoaxial rotation and transmit tension across the inferior MDB. This would also prevent dural enfolding at the level of the atlantoaxial interspace [4,9]. Through these mechanisms, the superior and inferior myodural communicating structures may influence the cerebrospinal fluid dynamics of the upper cervical region [9].

Failure of the bridging communications attaching onto the PAOM may cause various pathophysiological findings or neurological complications [29]. Micro-strains or trauma of the suboccipital muscles and tendons may cause clonus and generate cervicogenic headaches by stimulating the pain-sensitive dura via the PAOM [18]. This is further supported by a case report of chronic, intractable cervicogenic headaches relieved by the superior MDB’s surgical release [29]. The dural region, which indirectly attaches to the myodural connections, is innervated by nerves of the spinal trigeminal nucleus [30]. These bridging connections may be transmitting tension and generating nociceptive signals via this trigeminal sensory distribution [4,9]. Given that the dense connective tissue component of the meningovertebral ligaments attaches to the dura mater via the PAOM in the same manner as the myodural bridging structures, these ligaments may also be subject to similar pathologic complications such as spinal cord compression, cerebrospinal fluid accumulation, cervicogenic headaches, and trigeminal nerve pain distribution [9].

This study primarily used sagittal sheet plastination to investigate the anatomy of the PAOM. Confocal microscopy or histological studies would be a helpful resource when attempting to define details that construct the membranodural complex and its related structures. Coronal and transverse sheet plastination may further explain the PAOM’s layered anatomy and its relationship with dura and myovertebral structures that attach to it. The exact measurements (morphometrics) of these structures also warrant further investigation since the plastination process causes the shrinking of tissues.

## Conclusions

The results of this study clarify the intricate anatomy of the upper cervical region. Plastination reveals that the PAOM extends from the occiput and merges with the craniocervical dura mater forming a membranodural complex. The superior and inferior myodural, vertebrodural structures, and nuchal bridge all anchor into this posterior membranodural complex, which ends at the level of C3. Therefore, the median aspect of the PAOM does not directly attach to the posterior arch of the atlas. This morphology contradicts the traditional description of this membrane and explains how the superior MDB enters the epidural space. Defining the PAOM’s unique arrangement with plastination adds to the growing evidence that the myovertebrodural structures may prevent dural enfolding and influence cerebrospinal fluid dynamics at the cerebrospinal cistern. This anatomy is essential for physicians involved in head and neck pain and spine care as it may be affected by trauma, tumors, and congenital malformations. 
